# Pregnancy prediction in Nelore heifers using machine learning algorithms

**DOI:** 10.1007/s11250-026-05072-z

**Published:** 2026-05-12

**Authors:** Feliciano Benedetti de Freitas, Raimundo Nonato Colares Camargo Júnior, Welligton Conceição da Silva, Simone Inoe Araújo, Cláudio Vieira de Araújo

**Affiliations:** 1 Federal University of Mato Grosso, Sinop, Brazil; 2 Federal Institute of Pará (IFPA), Santarém, Pará Brazil

**Keywords:** Beef heifers, Machine learning, Pregnancy prediction, Reproductive efficiency

## Abstract

Beef cattle production systems, particularly those based on Nelore heifers in tropical regions, are under increasing pressure to improve reproductive efficiency while reducing production costs. Early identification of females with high reproductive potential remains a major challenge, especially under field conditions using routinely collected phenotypic data. This study aimed to develop and compare supervised machine learning models to predict pregnancy outcomes in Nelore heifers using growth-related traits. A dataset comprising 1,167 animals was used, including adjusted body weights at weaning (W210) and yearling (W365), average daily gain (DWG), and seasonal classification. Six algorithms were evaluated: Artificial Neural Networks (ANN), Random Forest (RF), Support Vector Machine (SVM), CatBoost, XGBoost, and LightGBM. Model performance was assessed using accuracy, F1-score, and the area under the receiver operating characteristic curve (AUC). The ANN achieved the highest accuracy (0.83), whereas RF showed the greatest discriminative ability (AUC = 0.94), followed by XGBoost and LightGBM (AUC = 0.93). In contrast, CatBoost exhibited low discriminative capacity (AUC = 0.53). Variable importance analysis consistently identified body weight at 210 days (W210) as the most influential predictor of pregnancy. These findings demonstrate that machine learning models can effectively support early and data-driven decision-making in beef cattle systems, enabling the identification of heifers with higher reproductive potential and reducing the maintenance of non-productive females. The use of easily obtainable growth traits reinforces the applicability of this approach, contributing to more efficient and sustainable reproductive management in tropical livestock production.

## Introduction

Beef cattle production plays a fundamental role in the global agricultural economy, particularly in tropical beef production systems dominated by Bos indicus breeds, where pasture-based systems predominate (Pandey and Upadhyay 2022). In these environments, improving reproductive efficiency is essential to increase productivity, reduce production costs, and enhance sustainability. Among the factors influencing herd performance, the reproductive success of replacement heifers is especially critical, as it directly affects herd renewal, system profitability, and the biological efficiency of production cycles (Harrison et al. 2020). Animals that reach sexual maturity earlier and achieve higher conception rates contribute to shortening the production cycle, increasing lifetime productivity, and optimizing resource use (Fernandes Júnior et al. 2022).

In contrast, heifers that fail to conceive at the beginning of the breeding season represent a significant economic burden, as they increase maintenance costs without contributing to productive output. However, the early identification of heifers with high reproductive potential remains a major challenge, as traditional approaches rely on phenotypic evaluation and, in some cases, genetic analyses, which are often time-consuming, costly, and difficult to implement under field conditions (Cole et al. 2025). In this context, improving the ability to predict reproductive outcomes has become a key objective in modern livestock systems.

Recent advances in data availability and computational methods have created new opportunities to improve decision-making in livestock systems (Morrone et al. 2022). In this context, supervised machine learning techniques have emerged as powerful tools for modeling complex and non-linear relationships among biological variables, enabling more accurate predictions of productive and reproductive outcomes (Soloshenkov et al. 2024; Curti et al. 2023). Unlike traditional statistical approaches, which often rely on linear assumptions and predefined interactions, machine learning algorithms can capture hidden patterns and high-order interactions among variables, improving predictive performance in complex biological systems.

Despite these advances, there is still a need for practical and cost-effective tools capable of predicting reproductive performance using routinely collected data in commercial herds. In tropical beef systems, where detailed reproductive records are often limited, growth-related traits such as body weight and weight gain may serve as indirect indicators of physiological development, nutritional status, and reproductive readiness. However, there is still a lack of studies evaluating the performance of machine learning models for predicting pregnancy in beef heifers under tropical production systems using routinely collected phenotypic data. Thus, integrating machine learning with easily obtainable phenotypic data represents a promising strategy to enhance reproductive management, optimize resource allocation, and improve system efficiency (Mikkola et al. 2024; Shine and Murphy 2022).

In this context, the aim of this study was to evaluate and compare the performance of different supervised machine learning algorithms: Artificial Neural Networks (ANN), Random Forest (RF), Support Vector Machine (SVM), CatBoost, XGBoost, and LightGBM—for predicting pregnancy outcomes in Nelore heifers based on growth-related traits. The results are expected to support early selection decisions and improve reproductive efficiency in tropical beef cattle production systems.

## Materials and methods

A dataset comprising pregnancy diagnosis records from 1,167 beef heifers was used, including 568 pregnant (48.7%) and 599 non-pregnant (51.3%) animals, indicating a balanced class distribution. Only heifers aged between 365 and 1,000 days were considered, ensuring that the analysis was conducted within a physiologically relevant age range for reproductive evaluation. The data were obtained from a commercial herd at Agropecuária Fogliatelli S/A, located at Porto do Campo Farm, in Lambari d’Oeste, Mato Grosso, Brazil. Animals were raised under a pasture-based production system with ad libitum mineral supplementation.

Body weights at weaning and at yearling were adjusted to 210 and 365 days of age, respectively, using simple linear regression. Weights were adjusted using the following equation: $\:{y}_{i}\left(ajusted\right)={y}_{i}+{\widehat{\beta\:}\:}_{1}(cte-iage)$, where $\:{y}_{i}\left(ajusted\right)$ and $\:{y}_{i}$ are the adjusted and observed body weights at weaning and at year; $\:{\widehat{\beta\:}\:}_{1}$ is the regression coefficient of weight as a function of age at weighing; cte is equal to 210 and 365 for adjusted weaning and yearling weights, respectively.

The daily weight gain (DWG) was calculated with the adjusted weaning weights (W210) and yearling weights (W365) based on the interval of 155 days between 210 and 365 days of age.

Based on the yearly weight collection date, the month of evaluation was determined using the yearling weight collection date, allowing the animals to be classified into two seasonal categories: season = 1: evaluations carried out between April and September, and season = 2: evaluations carried out between October and March. The response variable used was pregnancy diagnosis, indicating the presence (1) or absence (0) of confirmed pregnancy in the animals. The subsequent analyses considered this variable as a binary outcome.

The analytical approach was based on supervised binary classification, in which the response variable (Y) represented pregnancy status (1 = pregnant; 0 = non-pregnant). The models were trained to estimate the conditional probability P(Y = 1 | X), where X corresponds to the set of predictor variables: adjusted body weight at weaning (W210), adjusted body weight at yearling (W365), average daily gain (DWG), and season of evaluation. Each algorithm aimed to learn a function f(X) capable of accurately classifying animals and discriminating between pregnancy outcomes based on these input features.

Body weights at weaning and at yearling were adjusted to 210 and 365 days of age, respectively, using simple linear regression. Weights were adjusted using the following equation: $\:{y}_{i}\left(ajusted\right)={y}_{i}+{\widehat{\beta\:}\:}_{1}\left(cte-{age}_{i}\right)$, where $\:{y}_{i}\left(ajusted\right)$ and $\:{y}_{i}$ are the adjusted and observed body weights at weaning and at year; $\:{\widehat{\beta\:}\:}_{1}$ is the regression coefficient of weight as a function of age at weighing and yearling; cte is equal to 210 and 365 for adjusted weaning and yearling weights, respectively. Average daily gain (DWG) was calculated based on the difference between W210 and W365 over a 155-day interval.

Season of evaluation was defined based on the date of yearling weight measurement and categorized into two periods: dry season (April to September) and rainy season (October to March). The dataset was structured in Python using the pandas library. Predictor variables (X) and the response variable (Y) were separated for analysis. The data were randomly split into training (70%) and testing (30%) sets using a fixed random state (random_state = 42) to ensure reproducibility. Feature scaling was performed using the z-score standardization method (StandardScaler), with parameters estimated from the training set and applied to the test set. The descriptive statistics of the training and test samples are shown in Table 1.

**Table 1:** Number of records (N), mean and standard deviation estimates (SD) of adjusted body weights at 210 (W210) and 365 (W365) days of age, and daily weight gain (DWG) between these periods, stratified by sexual precocity class and data partition (Training/Test)

Sample			W365			W210			DWG	
	Class	*N*	Mean	SD		Mean	SD		Mean	SD
Training	0	413	297.78	47.69		225.14	27.66		0.469	0.241
	1	403	289.41	36.79		220.84	25.49		0.442	0.193
Test	0	186	298.89	40.57		224.53	23.63		0.480	0.213
	1	165	288.37	38.46		216.43	24.35		0.461	0.213

Six supervised machine learning algorithms were evaluated: Artificial Neural Networks (ANN), Random Forest (RF), Support Vector Machine (SVM), CatBoost, XGBoost, and LightGBM. The ANN model was implemented using the Keras Sequential API with a TensorFlow backend, employing ReLU activation functions in hidden layers and a sigmoid activation function in the output layer. Tree-based models (RF, XGBoost, LightGBM, and CatBoost) and the SVM were implemented using their respective Python libraries. Model architectures and parameters were defined to allow flexibility in capturing non-linear relationships among variables.

Hyperparameter optimization was performed using grid search with three-fold cross-validation (GridSearchCV) applied exclusively to the training dataset. Specific parameter grids were defined for each algorithm, including parameters such as number of estimators, tree depth, learning rate, regularization terms, kernel type, and network architecture. This approach ensured that model tuning was conducted without information leakage from the test dataset.

Model performance was evaluated on the independent test set using accuracy, precision, recall, F1-score, and the area under the receiver operating characteristic curve (AUC). These metrics were selected to provide a comprehensive assessment of both classification accuracy and discriminative ability. The use of an independent test set ensured an unbiased evaluation of model generalization.

The importance of predictor variables was assessed using permutation importance. This method evaluates the contribution of each variable by measuring the decrease in model performance after randomly shuffling its values. The resulting importance scores were used to identify the main phenotypic factors influencing pregnancy prediction.

## Results and discussion

The evaluated machine learning models demonstrated consistent performance in predicting pregnancy outcomes, with notable differences in their discriminative capacity. Table 2 summarizes the main performance metrics of the evaluated models. Overall, all algorithms, including Artificial Neural Networks (ANN), Random Forest (RF), XGBoost, LightGBM, CatBoost, and Support Vector Machine (SVM), showed satisfactory results in terms of accuracy, precision, recall, and F1-score. Despite the similarity in these metrics, relevant differences were observed in the models’ ability to discriminate between classes.

**Table 2:** Performance of machine learning models in predicting negative and positive pregnancies: precision, recall and F1-score

Algoritmo		Negative Pregnancies				Positive Pregnancies		
		Precision	Recall	F1-Score		Precision	Recall	F1-Score
ANN		0.79	0.84	0.82		0.86	0.82	0.84
model’s accuracy	0.83							
CatBoost		0.79	0.84	0.81		0.86	0.81	0.84
model’s accuracy	0.82							
XGBoost		0.78	0.84	0.81		0.86	0.81	0.83
model’s accuracy	0.82							
LightGBM		0.79	0.82	0.80		0.84	0.82	0.83
model’s accuracy	0.82							
SVM		0.79	0.78	0.79		0.82	0.83	0.83
model’s accuracy	0.81							
RF		0.77	0.83	0.8		0.85	0.8	0.82
model’s accuracy	0.81							

**Fig. 1:**
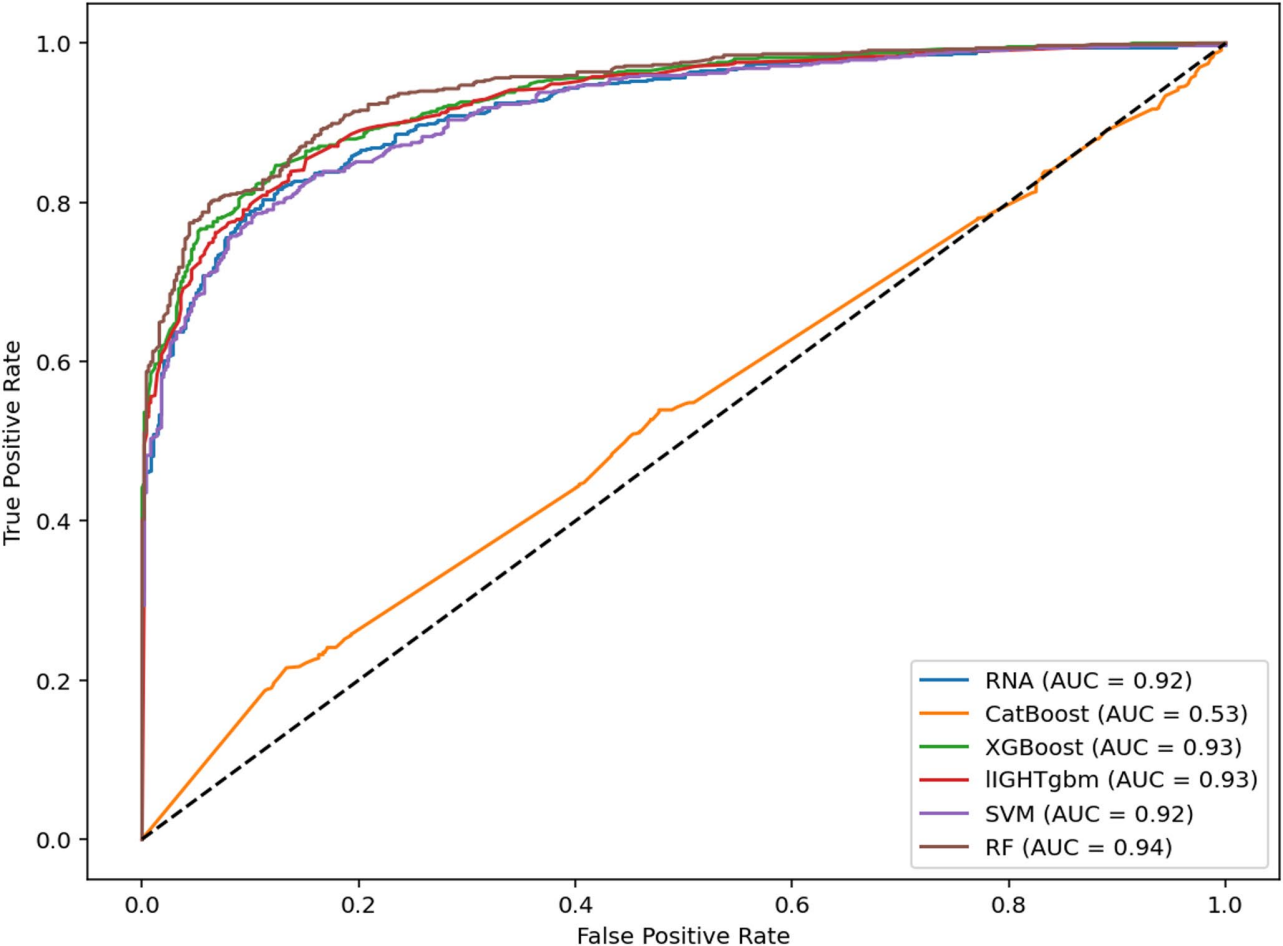
Comparative performance of machine learning models on the test dataset, evaluated by ROC curves (receiver operating characteristic) and AUC (area under the curve)

Despite the similarity between the accuracy and F1-score values presented in Table 2, the ROC curves revealed important differences in the discriminative performance of the models. Analysis of the area under the curve (AUC) showed that Random Forest (AUC = 0.94), XGBoost and LightGBM (AUC = 0.93), and Artificial Neural Network and SVM (AUC = 0.92) outperformed the CatBoost algorithm (AUC = 0.53), whose curve remained close to the baseline (AUC = 0.50), indicating an almost random classification behavior in this dataset (Fig. 1).

These results demonstrate that, although CatBoost achieved competitive performance in point metrics such as accuracy and F1-score, its low AUC indicates limited ability to correctly rank animals across different probability thresholds. This aspect is particularly critical in reproductive prediction scenarios, where minimizing false negatives is essential. The discrepancy between classification metrics and AUC has been widely discussed in the literature, highlighting that models may appear accurate while failing to provide reliable probabilistic discrimination due to calibration issues (Muschelli 2020; Niculescu-Mizil and Caruana 2005).

The reduced performance of CatBoost may be associated with the intrinsic characteristics of the dataset, which is predominantly composed of continuous variables (W210, W365, and DWG). CatBoost is specifically designed to handle categorical features through ordered boosting and target statistics (Prokhorenkova et al. 2018), and its efficiency may be limited when such structures are absent. Additionally, the lack of feature engineering strategies, such as the generation of synthetic attributes, may have further constrained its discriminative capacity, as reported by Wang and Cheng (2021). These findings reinforce that model performance is strongly influenced not only by algorithmic structure but also by the nature and informativeness of the input variables.

The interpretation of model performance should therefore be aligned with the intended application in beef production systems. Although the ANN achieved the highest accuracy (0.83), the Random Forest, XGBoost, and LightGBM models demonstrated superior global discriminative capacity, as reflected by their higher AUC values. This distinction is particularly relevant in contexts where flexible decision thresholds are required, such as identifying animals with lower reproductive potential for early culling. In such cases, models with higher AUC values provide more reliable probabilistic ranking, enabling more efficient allocation of resources. Conversely, when decisions are based on a fixed classification threshold, the ANN represents a consistent and robust alternative. Thus, model selection should consider the relative cost of classification errors and the specific objectives of the production system.

The robust performance of XGBoost and LightGBM observed in this study is consistent with previous findings that highlight their computational efficiency and strong generalization capacity in binary classification problems (Chen and Guestrin 2016). Similarly, the competitive performance of ANN corroborates studies demonstrating the ability of neural networks to capture complex and non-linear relationships in biological systems (LeCun et al. 2015).

Overall, all evaluated models, with the exception of CatBoost, proved suitable for predicting pregnancy outcomes, particularly in scenarios where reducing false negatives is a priority. These results emphasize that model selection should not rely solely on overall accuracy but must also consider discriminative capacity, as measured by AUC. The underperformance of CatBoost further illustrates how the interaction between algorithm design and dataset characteristics can influence predictive outcomes.

The predictive performance observed in this study should be interpreted within the context of the available input variables. The predictors used (W210, W365, daily weight gain, and season of evaluation) represent the phenotypic information consistently available in the farm database and were intentionally selected due to their practical applicability. These variables provide indirect but biologically meaningful indicators of nutritional and metabolic status, which are closely associated with physiological maturation and reproductive performance. However, additional reproductive variables, such as body condition score, precise age at breeding, and reproductive management strategies, were not available in a structured format for inclusion in the models.

It is important to note that reproductive management was standardized across the herd, as all animals were subjected to the same artificial insemination protocol and managed by the same technician. Furthermore, the production system followed a consistent pattern based on grazing, mineral supplementation, and protein supplementation during the dry season, which is typical of beef cattle systems in the Central-West region of Brazil. Therefore, the modeling approach intentionally relied on growth-related traits as biologically relevant proxies, while acknowledging that the inclusion of additional reproductive variables could further improve predictive performance.

Although the results presented in Table 2 indicate that ANN achieved the highest accuracy, the ROC curves (Fig. 1) demonstrate that Random Forest, XGBoost, and LightGBM exhibited slightly superior discriminative ability. Consequently, when the objective is to maximize classification accuracy at a fixed threshold, ANN represents an appropriate choice. However, when the goal is to optimize the overall discrimination between classes, particularly under varying decision thresholds, Random Forest emerges as the most robust alternative, followed by XGBoost and LightGBM.

**Fig. 2:**
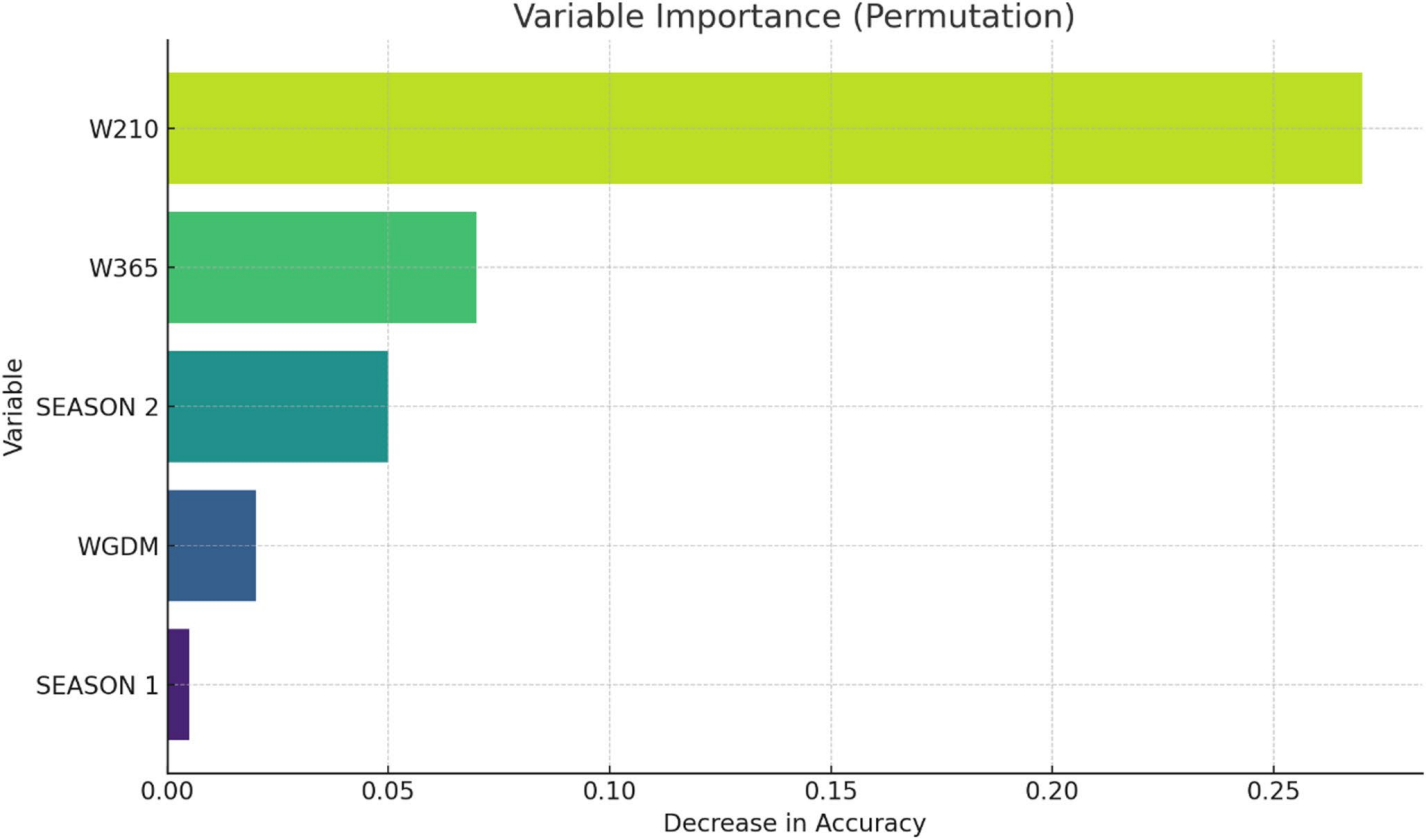
Relative importance of variables: body weight at 210 days (W210) and 365 days (W365) of age, weight gain during the dairy period (DWG), season 1, and season 2 in the classification of heifer pregnancy, assessed by permutation of accuracy in the ANN model

The analysis of variable importance, obtained through permutation of accuracy in the ANN model (Fig. 2), showed that body weight at 210 days (W210) was the most relevant predictor of pregnancy classification. The remaining variables, including body weight at 365 days (W365), season of evaluation, and daily weight gain (DWG), contributed less to model performance. The predominance of W210 is biologically consistent, as reproductive performance in beef heifers is closely associated with energy balance and metabolic status prior to the breeding season. Adequate growth supports endocrine function, including the secretion of gonadotrophic hormones such as luteinizing hormone and follicle-stimulating hormone, which are essential for follicular development and ovulation (Baumgaertner et al. 2024; Evans et al. 2022).

Conversely, animals with insufficient growth are more likely to present reproductive impairments, such as anestrus, silent ovulation, or irregular estrous cycles, which reduce conception rates (Frau et al. 2024; Nasution et al. 2021; Rademacher et al. 2015). These effects are mediated by metabolic signals such as insulin and leptin, which regulate energy availability and influence the hypothalamic–pituitary–gonadal axis (Athar et al. 2024; Desta 2024; Gong 2002; di Clemente et al. 2021; Zhang et al. 2023; Childs et al. 2020; Iwasa et al. 2022). Therefore, body weight can be interpreted as an integrative indicator of the physiological conditions required for pregnancy, reinforcing the importance of nutritional management strategies aimed at ensuring adequate growth during the rearing phase (Carvalho et al. 2022; Nazhat et al. 2021; Ding et al. 2024; Kasimanickam et al. 2021).

From a practical standpoint, the predictive models developed in this study can be directly applied to support herd management decisions. Producers can use predicted probabilities to classify heifers into different reproductive potential groups, enabling early culling of animals with low likelihood of pregnancy or their redirection to alternative production systems. Conversely, animals with higher predicted probabilities can be prioritized for nutritional and reproductive management, optimizing resource allocation and improving overall herd efficiency.

Methodologically, model robustness was assessed using a single 70/30 hold-out split. Although this approach allowed evaluation on an independent test set and demonstrated consistent performance between training and testing, it does not capture variability associated with different data partitions. Therefore, future studies may benefit from repeated or nested cross-validation to obtain more robust estimates of predictive performance. Even so, the consistency of the present results supports the biological relevance of growth-related traits as indicators of reproductive potential.

The influence of body weight on reproductive performance has been widely reported. Studies by Cooke et al. (2021) and Menegaz et al. (2008) demonstrated that heifers with higher weight gain during pre- and post-weaning phases exhibited higher pregnancy rates, corroborating findings from de Paula et al. (2022)d pez et al. (2018). Additionally, Ferreira et al. (2024), Pilau and Lobato (2009), and Silva et al. (2022) reported that nutritional supplementation during the reproductive period increases weight gain and the proportion of pregnant heifers. Montanholi et al. (2004) also observed a positive association between growth intensity during rearing and reproductive performance. These findings reinforce the importance of nutritional strategies aimed at ensuring adequate body development as a key factor for improving reproductive efficiency in beef heifers.

## Conclusion

This study demonstrates the applicability of machine learning approaches as effective tools for predicting pregnancy outcomes in beef heifers under tropical production conditions.

The findings highlight the relevance of growth-related traits as practical and biologically meaningful indicators of reproductive potential, reinforcing the importance of early-life nutritional management in improving reproductive efficiency.

The use of routinely collected phenotypic data allows for the early stratification of animals according to their reproductive potential, supporting more efficient decision-making and resource allocation in commercial herds.

Overall, this study provides a consistent and applicable framework for incorporating data-driven approaches into reproductive management, contributing to more efficient, sustainable, and precision-oriented beef cattle production systems.

## Data Availability

The datasets generated and analyzed during the current study are not publicly available due to commercial confidentiality but are available from the corresponding author on reasonable request.
